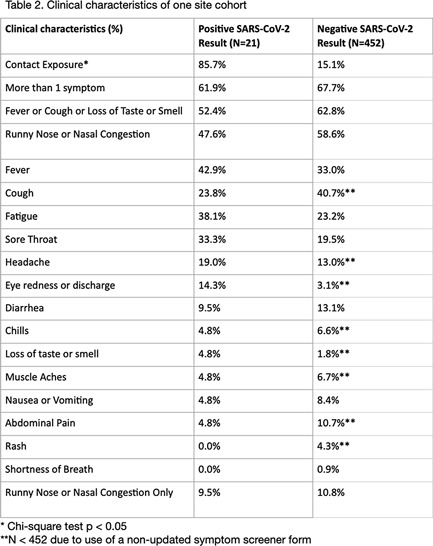# Optimizing COVID-19 Symptom Screening in the Pediatric Population

**DOI:** 10.1017/ash.2021.107

**Published:** 2021-07-29

**Authors:** Geena Zhou, Prachi Singh, Emily R. Perito, Naomi Bardach, Nicole Penwill, William Burrough, Ann Cheung, Margaret Nguyen, Shalini Mittal, Grace Cheng, Mia-Ashley Spad

## Abstract

**Background:** Research analyzing COVID-19 symptom screening has primarily focused on adult patients. In efforts to safely reopen schools, symptom screeners are being widely utilized. However, pediatric-specific outpatient data on which symptom combinations best identify children with COVID-19 are lacking. Such data could refine school symptom screening by improving screener sensitivity and specificity. In this study, we assessed the frequency of symptoms and symptom combinations in children tested for SARS-CoV-2 in outpatient settings. We aim to contribute to the optimization of pediatric COVID-19 screening questionnaires, to ultimately minimize both COVID-19 transmission in schools and missed school days. **Methods:** We conducted a retrospective analysis of outpatient symptoms screens, SARS-CoV-2 test results, and demographics of children (≤18 years) tested for SARS-CoV-2 between March 30 and November 30, 2020, at 3 UCSF-affiliated COVID-19 outpatient screening clinics in northern California. Those with incomplete symptom screens, >7 days between symptom documentation and test, and invalid test results were excluded. **Results:** Of 473 children tested at 1 site, 21 children had positive SARs-CoV-2 results and 452 children had negative results (4.4% positivity rate). Moreover, 85.7% of SARS-CoV-2–positive children had a known exposure to COVID-19 (Table 1). Of SARS-CoV-2–positive children, 61.9% had >1 symptom. Also, 52.4% of SARS-CoV-2–positive children had at least 1 symptom (fever, cough, or loss of taste or smell) versus 62.8% of SARS-CoV-2–negative children (Table 2). Runny nose or nasal congestion was the most frequently reported symptom in the SARS-CoV-2–positive group (47.6%) as well as the SARS-CoV-2–negative group (58.6%). Also, 14.3% of SARS-CoV-2–positive children had eye redness or discharge versus 3.1% of SARS-CoV-2–negative children. Isolated runny nose presented in 10.8% of SARS-CoV-2–negative versus 9.5% of SARS-CoV-2–positive children. All children with isolated diarrhea (n = 5), isolated headache (n = 3), and isolated rash (n = 2) tested negative. Preliminary symptom data based on 176 children from a second site showed that 9.9% of symptomatic children had a positive test result. **Conclusions:** Runny nose or nasal congestion was the most frequently reported symptom in all children tested for SARS-CoV-2. However, isolated runny nose or nasal congestion identified 2 cases of COVID-19 in our cohort. Eye redness or discharge may be an important symptom to screen for COVID-19 in children. Further research with a larger number of positive cases is needed to make conclusions about improving efficiency and efficacy of symptom screeners for COVID-19 in children.

**Funding:** No

**Disclosures:** None

**Table 1.**
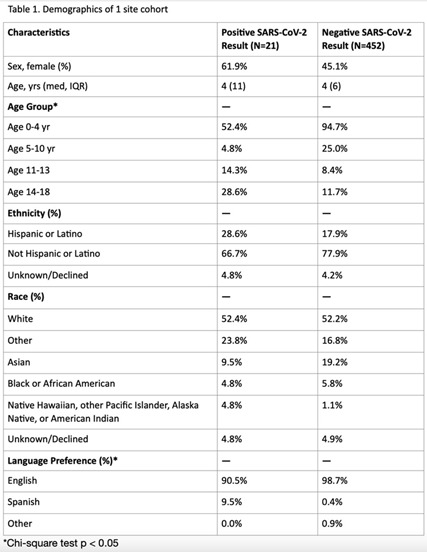

**Table 2.**